# VSIG4 Is Dispensable for Tumor Growth and Metastasis in Murine Colorectal and Breast Cancer Models

**DOI:** 10.3390/cancers17193207

**Published:** 2025-10-01

**Authors:** Els Lebegge, Neema Ahishakiye Jumapili, Jolien Van Craenenbroeck, Daliya Kancheva, Máté Kiss, Romina Mora Barthelmess, Ahmed E. I. Hamouda, Yvon Elkrim, Geert Raes, Éva Hadadi, Damya Laoui, Jo A. Van Ginderachter, Sana M. Arnouk

**Affiliations:** 1 Laboratory of Cellular and Molecular Immunology, Brussels Center for Immunology (BCIM), Vrije Universiteit Brussel, Pleinlaan 2, 1050 Brussels, Belgium; els.lebegge@vub.be (E.L.); ahishakiye.neema.jumapili@vub.be (N.A.J.); jolien.van.craenenbroeck@vub.be (J.V.C.); daliya.kancheva@vub.be (D.K.); mate.kiss@unige.ch (M.K.); romina.mora.barthelmess@vub.be (R.M.B.); ahmed.emad.ibrahim.hamouda@vub.be (A.E.I.H.); yvon.elkrim@vub.be (Y.E.); geert.raes@vub.be (G.R.); eva.hadadi@vub.be (É.H.); dlaoui@vub.be (D.L.); sana.arnouk@vub.be (S.M.A.); 2 Laboratory of Myeloid Cell Immunology, VIB Center for Inflammation Research, Pleinlaan 2, 1050 Brussels, Belgium; 3 Laboratory of Dendritic Cell Biology and Cancer Immunotherapy, VIB Center for Inflammation Research, Pleinlaan 2, 1050 Brussels, Belgium

**Keywords:** cancer, tumor microenvironment, colorectal carcinoma, triple negative breast cancer, tumor-associated macrophages, Kupffer cells, large peritoneal macrophages, VSIG4

## Abstract

Background: Tumor-associated macrophages (TAMs) are important contributors to tumor progression and metastasis. Therefore, the identification of molecules that mediate these cells’ tumor-promoting functions is highly warranted. VSIG4 has been proposed as a macrophage immune checkpoint. Hence, we aim to investigate this marker in preclinical models. Methods: Publicly available scRNAseq datasets of human colorectal (CRC) and triple-negative breast (TNBC) carcinomas and their murine counterparts were reanalyzed to investigate the expression of VSIG4 in the different TAM populations. Moreover, tumors were grown in *Vsig4*-deficient mice to evaluate the effect on primary tumor characteristics. Finally, since liver Kupffer cells and large peritoneal macrophages are at least partly VSIG4-high, and are implicated in metastasis to those organs, the dissemination of CRC cancer cells to those sites was assessed in the *Vsig4*-deficient mice. Results: We demonstrate that *VSIG4* expression in human CRC and TNBC is mostly restricted to TAMs, and that its expression correlates with a worse prognosis. However, a striking finding was that no *Vsig4* mRNA nor protein could be detected in the microenvironment of primary CRC and TNBC murine tumors, resulting in a similar tumor growth in wild type versus *Vsig4*-deficient mice. Moreover, no major differences were observed in metastatic tumor load in the liver and peritoneal cavity, apart from a reduced metastasis to the omentum in *Vsig4*-deficient animals. Conclusions: Murine cancer models are not suitable to investigate the role of VSIG4 in primary tumors and VSIG4 deficiency did not alter liver nor peritoneal cavity metastasis in murine models, with the exception of the omentum.

## Simple Summary

The role of some immune cell types in promoting tumor growth has become indisputable. A prime example of this is tumor-associated macrophages (TAMs). One protein that has been associated with tumor-promoting TAMs is VSIG4. In this study, we aim to further dissect the role of VSIG4 in tumor progression and metastasis. Analysis of publicly available datasets of human cancers confirmed the expression of VSIG4 mainly on TAMs. However, VSIG4 expression was absent in murine TAMs. Consequently, we observed no differences in primary tumor growth in mice that lack the VSIG4 receptor, compared to their normal counterparts. In tumor-bearing mice, VSIG4 could be detected on macrophages in healthy organs, but its absence did not affect metastasis to those organs. Therefore, murine models are not suitable to study the role of VSIG4 in TAMs and tissue macrophage-expressed VSIG4 does not seem to play an important role in metastasis in mice.

## 1. Introduction

Immunotherapy has witnessed a clinical breakthrough since the discovery of immune checkpoint blockers (ICB). Despite the successful therapeutic application of ICBs, most cancer patients are still refractory to this treatment. Resistance mechanisms to ICB have been described, including the presence of immune suppressive cells in the tumor microenvironment (TME) [1]. Tumor-associated macrophages (TAMs) are known to promote tumor progression and metastasis in cancer types such as colorectal carcinomas (CRC) [2,3] and triple-negative breast carcinomas (TNBC) [4], but also mediate resistance to ICB in those cancer types [5,6]. Therefore, there is a need for novel therapeutic TAM markers that can alleviate TAM-induced immune suppression and therapy resistance.

VSIG4, a member of the B7 protein family, was recently suggested as a novel immune checkpoint molecule [7]. In steady state, VSIG4 is expressed by tissue macrophages, like resident peritoneal macrophages and liver Kupffer cells (KCs) [8,9]. VSIG4 has been suggested as a complement and pattern recognition receptor [10,11]. Upon ligand binding, the receptor is internalized, leading to phagocytosis [8,12] and to the suppression of proinflammatory macrophage activation [13]. In addition to its functions as an innate receptor, the VSIG4 receptor also binds CD8^+^ and CD4^+^ T cells and suppresses their proliferation and activation [7,14]. Moreover, computational analyses in CRC have hinted towards an immunoregulatory role for VSIG4 in macrophages [15]. Since VSIG4 plays a role in both innate and adaptive immunity, by targeting the VSIG4 receptor in cancer, the macrophage activation status could potentially be modulated from a trophic phenotype to an antitumoral phenotype, and VSIG4-mediated suppression of the antitumoral adaptive immune response could be reduced. For this reason, we investigated the role of the VSIG4 receptor on TAMs in solid tumors and assessed the possibility to use VSIG4 as a potential marker for TAM-directed therapeutic targeting.

The VSIG4 receptor presented itself as an interesting TAM marker in human CRC and TNBC, with a dominant expression on the FOLR2^+^ TAM subset. Importantly, VSIG4 expression associated with a worse prognosis in these malignancies [16,17,18,19,20]. Conversely, VSIG4 was dispensable for the progression of murine models of CRC and TNBC. In these models, TAMs did not express VSIG4 at the surface, nor was VSIG4 released as a soluble protein, and consequently, primary tumor growth was unaltered in WT versus VSIG4 knockout (KO) mice. Furthermore, in the liver and peritoneal cavity, where a resident VSIG4-expressing macrophage population is present, susceptibility to metastasis by colorectal cancer cells was similar in VSIG4 KO and WT mice.

## 2. Materials and Methods

### 2.1. Mice, Cell Culture and Tumor Models

**Figure 1:**
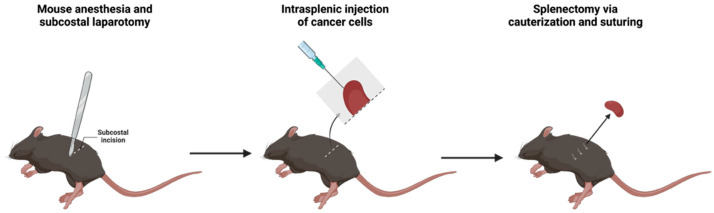
Liver metastasis model via intrasplenic injection followed by a splenectomy.

VSIG4 KO mice in C57Bl6 background were generously provided by Genentech (South San Francisco, CA, USA) and were bred in-house and VSIG4 WT littermates were used as controls. 6 to 12-week old female mice weighing between 17 and 22 g were used in experiments. Mice were maintained at the VUB animal facility with a standard room temperature (20 and 24 °C) and humidity (45 to 65%) and a 12 h light/dark cycle. The MC38 cell line was kindly provided by Massimiliano Mazzone (VIB-KULeuven, Leuven, Belgium). The MC38-Thy1.1 cell line was generated from the parental MC38 cell line as previously described [21]. E0771 cells were purchased from ATCC (Manassas, VA, USA). The MC38, MC38-Thy1.1 and E0771 cell line were cultured at 37 °C and 5% CO_2_ in DMEM supplemented with Pen/Strep, 10% FCS and glutamine. One million MC38 cancer cells were injected subcutaneously in 100 µL HBSS. 5 × 10^5^ E0771 cells were injected in the fourth inguinal mammary fat pad, resuspended in 50 µL 50% (*v*/*v*) growth-factor reduced Matrigel (Corning)/HBSS. To mimic peritoneal metastasis of colorectal cancer cells, one million MC38-Thy1.1 cells were injected intraperitoneally in 200 µL HBSS. To obtain liver metastasis, two million MC38-Thy1.1 cancer cells were injected intrasplenic using a protocol adapted from O’ Brien et al. [22] (Figure 1). Mice were anesthetized with ketamine (100 mg/kg) and xylazine (10 mg/kg). A subcostal laparotomy was performed. Subsequently, cancer cells were slowly injected in a volume of 100 µL saline solution in the spleen tissue, after which a splenectomy was performed via cauterization.

Inclusion and exclusion criteria: All criteria were defined prior to the start of experiments. Mice would be excluded from the study when humane endpoints are reached prior to the predefined experimental endpoint. These humane endpoints follow the guidelines of the Belgian Council for Laboratory Animal Science and were approved by the Ethical Committee for Animal Experiments of the VUB and take into consideration the animal behavior, physical appearance, and tumor volume/ulceration. Moreover, tumor samples would be excluded when lymph node contamination is identified based on the abundances of B cells and naïve T cells. Statistical outliers would be identified with Rout test and excluded. No exclusions were carried out.

Randomization: in most cases, mouse allocation into the compared groups had to be performed based on their genotype. Otherwise, randomization was not performed. For tumor cell inoculation, mice from the WT and KO groups were injected in an alternating sequence (i.e., one WT mouse followed by one KO mouse) until all animals had been injected. This approach was used to minimize potential bias related to temporal variations in cell preparation or injection conditions.

Blinding: Throughout the experiment, animal care takers and the technician measuring tumor growth/mouse weight and sacrificing animals were blinded to the allocations of the groups. Data analysis was performed blinded to the groups.

### 2.2. Ex Vivo Preparation of Single-Cell Suspensions

The orthotopic E0771 tumors, subcutaneous MC38 tumors and omental MC38-Thy1.1 tumors were processed as previously described [23]. In short, tumors were excised and cut in small pieces using scissors. The tumors were then enzymatically digested at 37 °C for 25 min in RMPI medium with 10 units/mL collagenase I, 400 units/mL collagenase IV and 30 units/mL deoxyribonuclease (DNAse)-I (Worthington, Lakewood, NJ, USA). The samples were then squashed and filtered in RMPI. Subsequently erythrocyte lysis was performed. Single-cell suspensions were kept on ice until further use.

Livers were processed as previously described [24]. Briefly, livers were perfused with 20 mL HBSS. Subsequently, livers were cut in RMPI buffer containing Collagenase A (5 mg/mL, Roche, Basel, Switzerland) and DNAse I (10 U/mL, Merck, Rahway, NJ, USA). Using a GentleMACS Dissociator (Miltenyi Biotech, Bergisch Gladbach, Germany) the liver suspensions were processed and incubated for 20 min at 37 °C. Livers were then placed on ice and processed again using the GentleMACS Dissociator. Next, the liver samples were filtered through a 100 µm filter and spun down for 6 min at 450 G and 4 °C. Red blood cell lysis was performed prior to antibody staining. Peritoneal exudate cells (PECs) were collected by collecting a peritoneal lavage with 10 mL ice-cold HBSS. In tumor-bearing animals, red blood cell lysis was performed. Subsequently, PECs were spun down and resuspended in HBSS for further processing.

### 2.3. Flow Cytometry

Once a single-cell suspension was obtained, dead cells were stained using a viability: Fixable Viability Stain 575V (1:2000, BD Biosciences, San Jose, CA, USA) or Fixable Viability Dye eFluor 506 (1:1000, eBioscience, San Diego, CA, USA). The PECs were washed with FACS buffer (HBSS enriched with 1% (*v*/*v*) FCS and 2mM EDTA) after 30 min of live/dead staining at 4 °C in the dark. Subsequently, Fcγ receptors were blocked for 15 min at 4 °C using an anti-CD16/CD32 antibody (clone 2.4G2, in-house). The single-cell suspensions were stained with fluorophore-conjugated Abs diluted in FACS buffer (20 min at 4 °C in the dark). A summary of all used Abs is present in Table S1. Flow cytometry data was acquired using the BD FACSCantoII and FACSSymphony A3 and analyzed using FlowJo (TreeStar, Inc., Ashland, OR, USA)). Gating strategies to identify immune cell populations are shown in Figures S5, S7 and S8.

### 2.4. Sandwich ELISA

The anti-VSIG4 Nb was produced in bacteria and purified via size exclusion chromatography as described elsewhere [25,26]. The anti-VSIG4 Nb was coated at 1 µg/mL on a 96-well adsorption immunoassay plate in PBS overnight at 4 °C. The next day, the plate was washed 3× with 0.05% PBS-Tween (R&D Systems, Minneapolis, MN, USA) and dried on paper towels after the last wash. The plate was then blocked with 200 µL 1% PBS-BSA for 1 h at 37 °C, covered with parafilm. The plate was again washed 3× with PBS-Tween. Subsequently, the tumor supernatant or recombinant mouse VSIG4 (50187-M08H, Sino biological, Eschborn, Germany) was added to the wells for 1 h at 37 °C. After incubation, the plate was washed 3× with PBS-Tween and incubated with the detecting anti-VSIG4 monoclonal antibody (1:2000, EPR22576-70, Abcam, Cambridge, UK) for 1 h at 37 °C. After 3× washing with PBS-Tween, the secondary goat anti-rabbit HRP-conjugated Ab (1:2000, CLAS09-605, Bethyl Laboratories, Huissen, The Netherlands) was added to the plate and incubated for 1 h at 37 °C. After 3 washing steps with PBS-Tween, the colorimetric reaction was performed by adding tetramethylbenzidine and hydrogen peroxide reaction to the wells at a 1:1 ratio in a final volume of 100 µL. After approximately 30 min of incubation in the dark, the reaction was stopped by adding 50 µL 1 M sulfuric acid to each well.

### 2.5. SDS-PAGE

10 ug of the positive control or 19.5 µL of each tumor supernatant was mixed with 7.5 µL NuPAGE^®^ LDS Sample buffer (4×) (Life Technologies, Carlsbad, CA, USA) and 3 µL NuPage^®^ sample reducing agent (10×) (Life Technologies, Carlsbad, CA, USA) to make a total of 30 µL mixture. Afterwards, the mixture was heated to a temperature between 70 and 100 °C for 10 min (Type 16500 heating block, Thermolyne, Bensonville, IL, USA) and loaded on a 12% NuPAGE^®^ Bis-Tris polyacrylamide gel (Life Technologies, Carlsbad, CA, USA). 5 µL of PageRuler Prestained Protein Ladder (ThermoFisher, Waltham, MA, USA) was used to define the molecular masses. The electrophoresis was performed in 1× MES buffer (Duchefa Biochemie, Haarlem, The Netherlands) at 100 Volts for approximately 1 h. Then, the gel was stained with coomassie blue for 2 h followed by O/N incubation in destaining solution at room temperature shaking, to visualize the stained proteins.

### 2.6. Western Blot

The 12% Bis/Tris gel was transferred onto a nitrocellulose membrane (GE Healthcare, Chicago, IL, USA) in 20% methanol, 3 g/L Trizma base (Sigma Aldrich, St. Louis, MO, USA) and 14.4 mL glycerine (Acros Organics, Geel, Belgium) for 1 h at 100 Volts and 250 mA. The blot was blocked for 1 h at room temperature with 2.5% *w*/*v* casein in PBS (pH 7.5). Subsequently, the blot was washed with 0.05% PBS-Tween (R&D Systems, Minneapolis, MN, USA). The blot was then incubated with anti-VSIG4 monoclonal antibody (1:2000, EPR22576-70, Abcam, Cambridge, UK) for 1 h at room temperature. After washing the blot with PBS-Tween, the blot was incubated with secondary goat anti-rabbit HRP-conjugated Ab (1:2000, CLAS09-605, Bethyl Laboratories, Huissen, The Netherlands) and an HRP-conjugated anti-His mAb (3D5, Thermofisher, Waltham, MA, USA). Next, the blot was developed by combining 4-chloro-1-naphtol (Sigma Aldrich, St. Louis, MO, USA) with 6 mL methanol (Acros Organics, Geel, Belgium), 30 mL TPA buffer and 18 µL hydrogen peroxide (Merck, Rahway, NJ, USA). The blot was developed overnight at 4 °C.

### 2.7. In Silico Analysis Human CRC and TNBC: scRNA-Sequencing Dataset and Correlation Analysis

Dataset was retrieved from Qian et al. [27]. The expression matrix was processed using Seurat v.3.2.2. Low-abundance genes were removed based on the distribution of the mean counts per gene for each sample, following the proposed Bioconductor workflow 3. The myeloid cells were extracted based on the “CellType” annotation in the cellular metadata provided by the authors, followed by library size normalization, highly variable gene selection, scaling and PCA using Seurat, batch correction across patients with harmony v.0.1.1, and UMAP and Louvain clustering. The cell type of each cluster was assigned based on differential gene expression analysis and the expression of specific cell type and functional gene markers. The proliferating cells, DCs and monocytes-macrophages, were individually further subsetted and re-clustered following the same procedure as above (highly variable gene selection, scaling, PCA, batch correction and Louvain clustering). Identified doublet cells, showing marker expression, specific for two different cell types were excluded from further analysis. Finally, all TAMs were subsetted and reclustered as described above.

Correlation analysis of VSIG4 expression in tumors of CRC patients was performed using http://kmplot.com.

### 2.8. Statistics

Data is presented as the mean ± standard error of the mean (SEM). GraphPad Prism 9.5.1 was used to calculate statistical significance. For pairwise comparisons, unpaired two-tailed Student’s *t*-test was performed. For the comparison of one or multiple groups, either a one-way analysis of variance (ANOVA) was performed with Tukey multiple comparisons test or a two-way ANOVA with Sidak multiple comparisons test was performed. The *p*-value is shown as such: * *p* ≤ 0.05, ** *p* ≤ 0.01, *** *p* ≤ 0.001, **** *p* ≤ 0.0001.

## 3. Results

### 3.1. VSIG4 Is Expressed by Human TNBC and CRC TAM Subsets and Correlates with a Worse Prognosis

**Figure 2:**
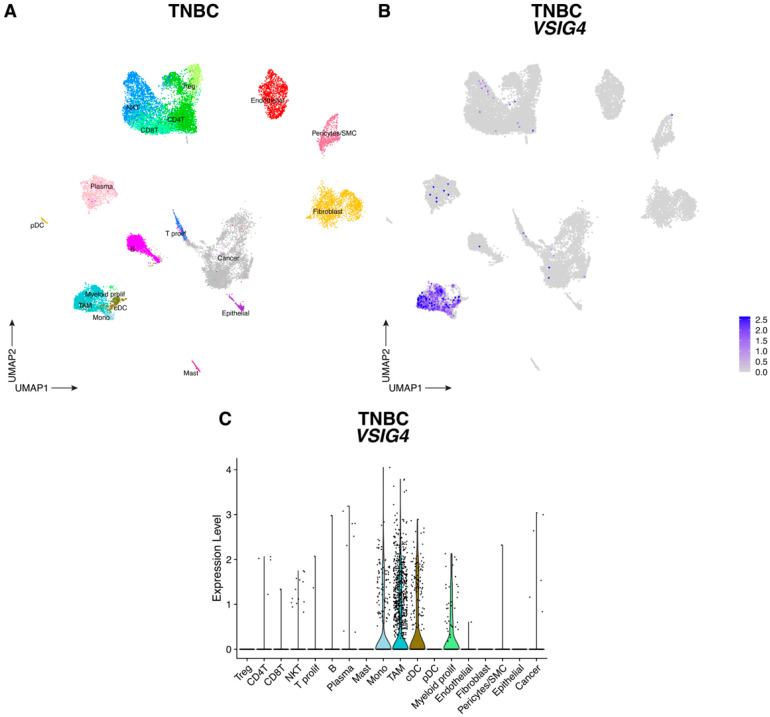
VSIG4 is expressed by myeloid cells in TNBC tumors. (**A**) UMAP plot of scRNAseq data showing 20,961 cells from TNBC tumors where the different cell types are assigned different colors. (**B**) UMAP feature plot of cells infiltrating TNBC tumors where *VSIG4* expression is indicated by the color. (**C**) Violin plots showing the expression of *VSIG4* in each cluster infiltrating TNBC tumors.

**Figure 3:**
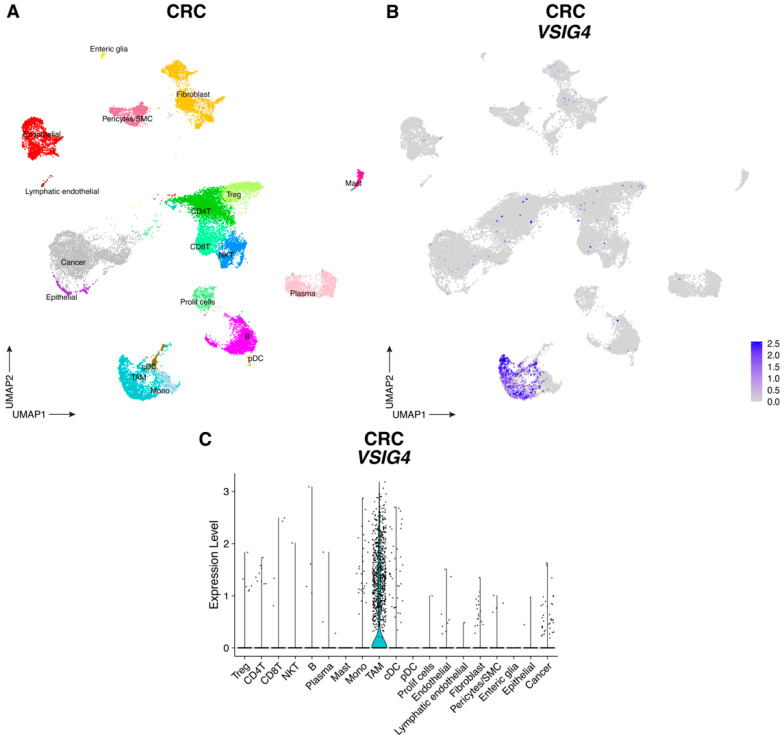
VSIG4 is expressed by TAMs in CRC tumors. (**A**) UMAP plot of scRNAseq data showing 24,521 cells from CRC tumors where the different cell types are assigned different colors. (**B**) UMAP feature plot of cells of infiltrating CRC tumors where *VSIG4* expression is indicated by the color. (**C**) Violin plots showing the expression of *VSIG4* in each cluster infiltrating CRC tumors.

Since VSIG4 is expressed by macrophages [7,8] and is known to suppress T-cell proliferation and activation, we hypothesized that VSIG4 on TAMs might contribute to immune suppression in the TME. First, we mined publicly available single cell RNA sequencing (scRNAseq) datasets encompassing all cell types from CRC and TNBC tumors [27], tumor types for which immunotherapy is promising but still too inefficient [28,29]. In both tumor types, *VSIG4* expression was restricted to the myeloid cell compartment. In TNBC tumors, *VSIG4* expression was observed in TAMs (*C1QA*, *C1QB*, *APOE*), monocytes (*S100A8*, *FCN1*, *VCAN*), proliferating myeloid cells (*C1QA*, *C1QB*, *MKI67*, *STMN1*) and cDC (*FCER1A*, *CD1C*) (Figure 2 and Figure S1A); while only macrophages were *VSIG4*-positive in CRC tumors (Figure 3 and Figure S1B).

**Figure 4:**
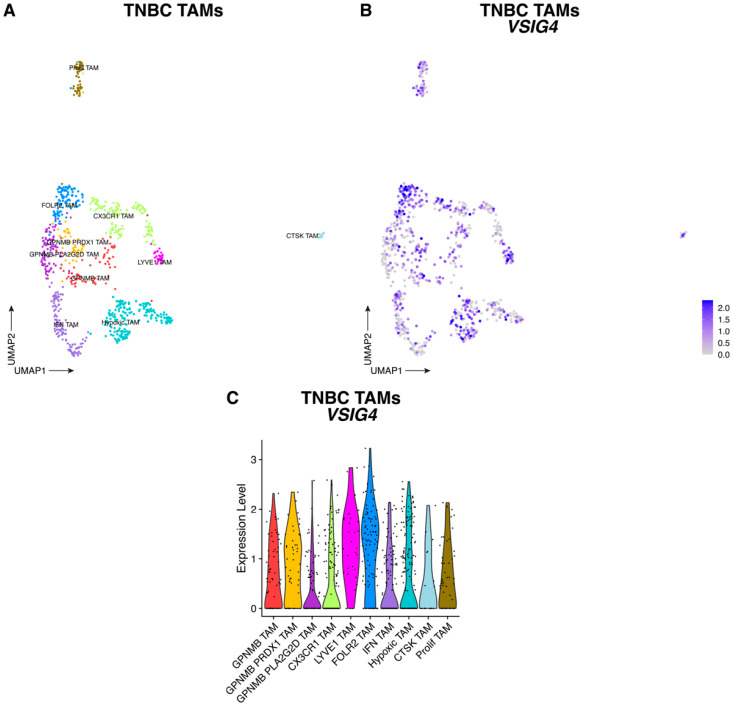
VSIG4 is expressed by TAMs infiltrating TNBC tumors. (**A**) UMAP plot of scRNAseq data showing 846 TAMs from TNBC tumors where the different TAM subsets are assigned different colors. (**B**) UMAP feature plot of TAMs infiltrating TNBC tumors where *VSIG4* expression level is indicated by the color. (**C**) Violin plots showing the expression of *VSIG4* in each TAM cluster infiltrating TNBC tumors.

**Figure 5:**
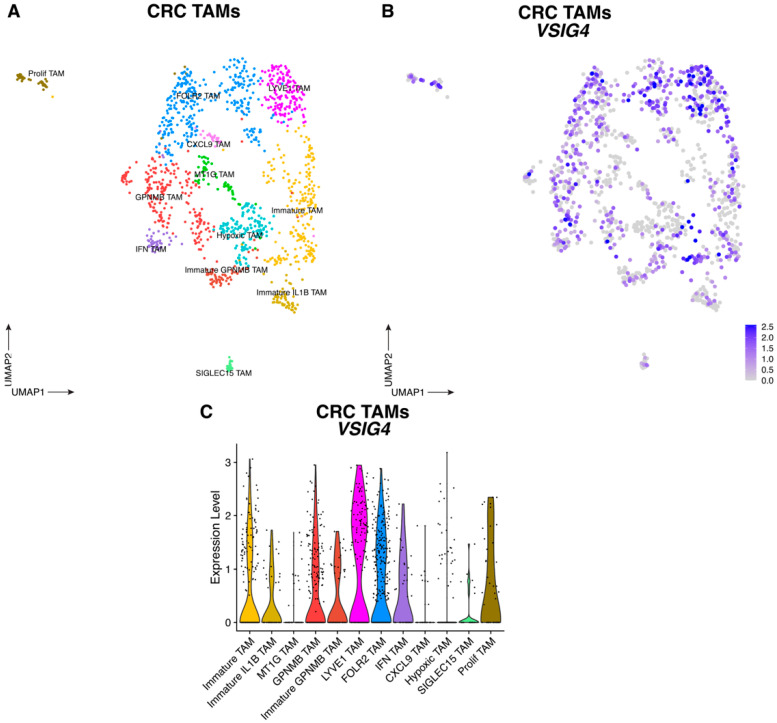
VSIG4 is expressed by TAMs infiltrating CRC tumors. (**A**) UMAP plot of scRNAseq data showing 1309 TAMs from CRC tumors where the different TAM subsets are assigned different colors. (**B**) UMAP feature plot of TAMs infiltrating CRC tumors where *VSIG4* expression is indicated by the color. (**C**) Violin plots showing the expression of *VSIG4* in each TAM cluster infiltrating CRC tumors. UMAP plot of scRNAseq data showing 20,961 cells from TNBC tumors.

Upon reclustering of macrophages, several TAM subsets were observed in both tumor types, all of which express *VSIG4* mRNA to some extent (Figure 4, Figure 5 and Figure S2). The highest *VSIG4* expressors in TNBC tumors were the LYVE1^+^ (*VCAN*, *LYVE1*, *EDNRB*, *CD163*) and FOLR2^+^ (*STAB1*, *SEPP1*, *FOLR2*, *IGF1*) TAM clusters (Figure 4 and Figure S2A). Accordingly, in CRC tumors, *VSIG4* expression was highest in LYVE1^+^ TAMs (which are also *FOLR2*-positive, and express the marker genes *CD163*, *SEPP1*, *F13A1*, *STAB1*) and FOLR2^+^ TAMs (which are *LYVE1*-negative, and express the marker gene *SERPINF1*), the latter expressing *CD163*, *SEPP1*, *F13A1* and *STAB1* at a somewhat lower level (Figure 5 and Figure S2B). These VSIG4-expressing TAMs likely contribute to tumor progression in both tumor types. Indeed, when employing the TCGA public database to subdivide CRC and TNBC tumor-bearing patients into those that express high or low levels of *VSIG4* in the TME, high *VSIG4* expression correlates with a worse prognosis only reaching significance for CRC (Figure S3). Since *VSIG4* expression is mostly restricted to macrophages in the TME, these data are strongly indicative of the tumor-promoting potential of VSIG4^+^ TAMs in these tumors.

### 3.2. The VSIG4 Protein Is Absent from Mouse Primary Tumors and, Consequently, Does Not Influence Tumor Growth

**Figure 6:**
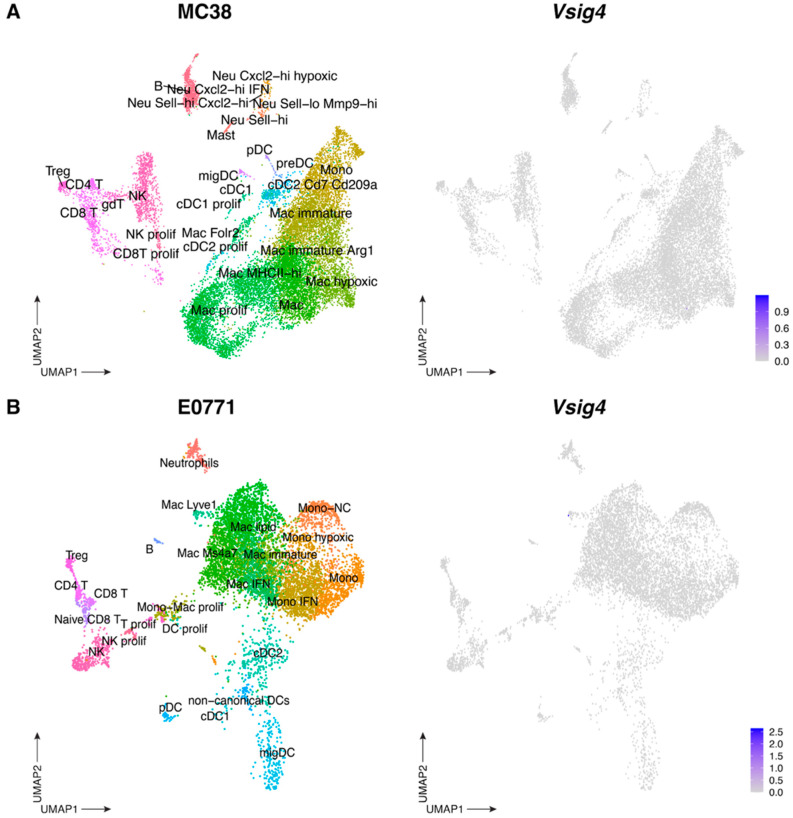
VSIG4 is not expressed by TAMs in the TME of murine tumors. (**A**) UMAP plot of CITEseq data showing 15,420 CD45^+^ cells from MC38 tumors where the different cell types are assigned different colors (**left**) and UMAP feature plot of cells infiltrating MC38 tumors where *Vsig4* expression is indicated by the color (**right**) (**B**) UMAP plot of CITEseq data showing 8489 CD45^+^ cells from E0771 tumors where the different cell types are assigned different colors (**left**) and UMAP feature plot of cells infiltrating E0771 where *Vsig4* expression is indicated by the color (**right**).

To elucidate the function of the VSIG4 receptor in the TME, we turned to mouse models of CRC (subcutaneous MC38) and TNBC (orthotopic E0771, grown in the mammary fat pad). Remarkably, cellular indexing of transcriptomes and epitopes by sequencing (CITEseq) analysis of MC38 [30] and E0771 [31] (in house generated datasets) tumors failed to detect significant levels of *Vsig4* mRNA in the CD45^+^ hematopoietic compartment, despite the presence of various TAM clusters (Figure 6 and Figure S4). As scRNAseq may fail to detect low abundant transcripts, we next verified whether the VSIG4 protein was expressed in these tumors. E0771 tumor single-cell suspensions were stained with anti-VSIG4 mAb, either extracellularly or intracellularly (all used Abs and the gating strategy are shown in Table S1 and Figure S5, respectively), but the VSIG4 protein could not be detected on the surface, nor within TAMs or any other live cells in these tumors (Figure S6A). Therefore, immune, stromal nor cancer cells express the VSIG4 receptor at the surface or in the cytosol in these murine tumors.

To assess whether the absence of the VSIG4 protein was a general phenomenon in tumor-bearing animals, we stained large peritoneal macrophages (LPMs) and liver KCs from tumor-bearing and naïve WT mice, as both of these macrophage populations are known to express VSIG4 at their surface [8,10,32,33]. The abundance of VSIG4-expressing LPMs and KCs was similar in tumor-bearing compared to naive WT mice (Figure S6B), illustrating that VSIG4 receptor expression is maintained on tissue-resident macrophages from tumor-bearing animals, but is absent from monocyte-derived TAMs in the TME.

**Figure 7:**
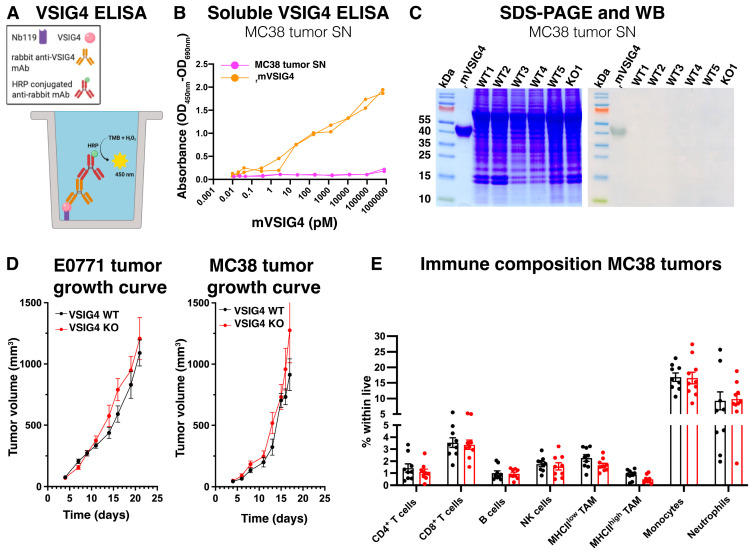
VSIG4 is absent from MC38 tumor supernatant and does not influence tumor characteristics. (**A**) Schematic representation of in-house VSIG4 sandwich ELISA. (**B**) An anti-VSIG4 Nb was coated as a capturing protein and a commercially available anti-VSIG4 mAb was used as a detecting protein. A horseradish peroxidase and tetramethylbenzidine colorimetric reaction in the presence of hydrogen peroxide was performed to assess the presence of soluble VSIG4 in MC38 tumor supernatant. The colorimetric reaction was measured at 450 nm and background subtraction at 690 nm was performed. Recombinant mouse VSIG4 (_r_mVSIG4) was used as a positive control. (**C**) SDS-PAGE (**left**) and Western blot (**right**) analysis of MC38 tumor supernatants of VSIG4 WT mice (lane 2–6). Recombinant mouse VSIG4 (_r_mVSIG4) was used as a positive control (lane 1) and the tumor supernatant of a VSIG4 KO mouse was used as a negative control (lane 7), uncropped blots (Figure S7). (**D**) Tumor growth curves of orthotopic E0771 and subcutaneous MC38 in VSIG4 WT (*n* = 9, 9) and VSIG4 KO (*n* = 9, 10) mice. (**E**) Percentage of immune population within all live cells of subcutaneous MC38 tumors in VSIG4 WT (*n* = 9) and VSIG4 KO (*n* = 10) mice.

Several studies have shown that VSIG4 can be released as a soluble protein and serves as a prognostic and/or therapeutic marker for ovarian cancer [20], lymphoma-associated lymphohistiocytosis [18], endometriosis [34] and bacterial peritonitis [35]. Since VSIG4 was not detected on the surface or in the cytosol of any cell within the TME, we investigated the possibility that VSIG4 was efficiently released as a soluble protein by these cells. Prior to adding tissue digesting enzymes, medium was collected from finely cut MC38 tumors (tumor supernatants) and soluble VSIG4 was detected using an in-house developed sandwich ELISA, employing our previously generated anti-VSIG4 capturing Nb [36] and a commercially available monoclonal detecting anti-VSIG4 antibody (Ab) (Figure 7A). While recombinant mouse VSIG4 (rmVSIG4) was efficiently detected by the sandwich ELISA, no VSIG4 could be found in MC38 tumor supernatants (Figure 7B). The tumor supernatants and rmVSIG4 were then loaded on an SDS-PAGE, with only the latter being detected via Western blot (Figure 7C). Altogether, these data demonstrate that VSIG4 is absent as a surface receptor, as an endosomal recycled receptor [8] and as a soluble protein in the TME of the investigated mouse solid tumor models. Consequently, no significant differences in tumor growth were observed between VSIG4 KO and VSIG4 WT littermate mice in either tumor model (Figure 7D). Moreover, the immune composition of the MC38 tumors was similar in both genotypes, demonstrating that VSIG4 is redundant for primary tumor progression and immune-related functions in the TME (Figure 7E).

### 3.3. The VSIG4 Receptor Does Not Play a Role in Metastatic Growth of Cancer Cells in the Peritoneal Cavity and Liver but Could Play a Role in the Omentum

**Figure 8:**
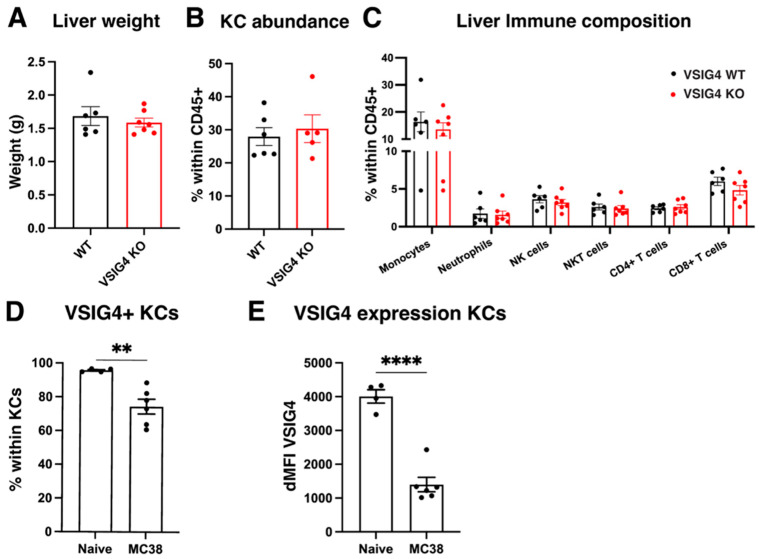
VSIG4 does not affect metastasis of MC38 cancer cells to the liver. (**A**) Liver weights of MC38 metastasis-bearing VSIG4 KO (*n* = 7) and WT (*n* = 6) mice at 10 days post-inoculation. (**B**) Percentage of KCs within all leukocytes in livers of MC38 metastasis-bearing VSIG4 KO (*n* = 5) and WT (*n* = 6) mice. (**C**) Percentage of immune populations within all leukocytes in livers of MC38 metastasis-bearing VSIG4 KO (*n* = 7) and WT (*n* = 5) mice. (**D**) Percentage of VSIG4^+^ KCs within all KCs in MC38 metastasis-bearing VSIG4 WT mice (*n* = 6) and naive VSIG4 WT mice (*n* = 4). (**E**) dMFI of VSIG4 within VSIG4^+^ KC in MC38 metastasis-bearing VSIG4 WT (*n* = 5) and naive VSIG4 WT (*n* = 4) mice. The dMFI is calculated by subtracting the average MFI of VSIG4 KO mice. ** *p* ≤ 0.01, **** *p* ≤ 0.0001.

We then assessed tumor progression in anatomical locations where a resident VSIG4^+^ macrophage population is present, such as the peritoneal cavity and the liver. As KCs are located in the liver sinusoids, where they aid in clearing blood borne pathogens, we opted for a cancer metastasis model, reasoning that KCs would be among the first cells to encounter incoming metastatic cancer cells [37,38]. An experimental model of colorectal cancer metastasis to the liver was performed in VSIG4 KO and WT mice through the intrasplenic injection of MC38 cancer cells. At 10 days post-injection (end point), we observed no significant differences in liver weight, suggesting that the metastatic burden in VSIG4 KO and WT mice was similar (Figure 8A). The percentage of KCs within all immune cells in the liver was also similar in VSIG4 KO and WT mice at 10 days post-injection (Figure 8B) and no differences in the liver immune composition were observed between VSIG4 KO and WT mice (Figure 8C, gating strategy in Figure S8). However, at 10 days post-injection in WT mice, the percentage of VSIG4^+^ KCs and their VSIG4 expression was lower compared to livers in naïve mice (Figure 8D,E). Overall, VSIG4 expression does not affect the susceptibility to liver metastasis and does not influence the liver immune composition upon cancer cell metastasis.

**Figure 9:**
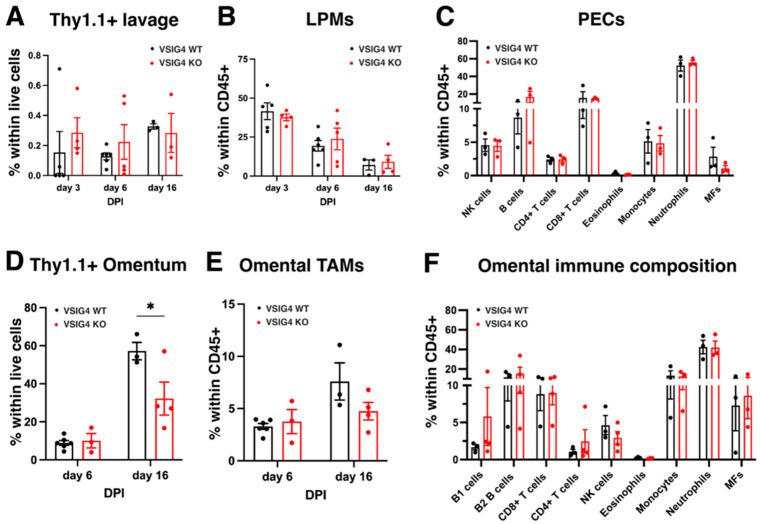
VSIG4 is not essential for the metastasis of MC38 cancer cells in the peritoneal cavity but possibly supports metastasis to the omentum. (**A**) Percentage of MC38 Thy1.1^+^ cancer cells in peritoneal lavages of VSIG4 KO and WT mice at day 3 (*n* = 4.5), day 6 (*n* = 5.6) and day 16 (*n* = 4.3) post-injection of MC38-Thy1.1 cancer cells. (**B**) Percentage of LPMs within all leukocytes in peritoneal lavages of MC38 metastasis-bearing VSIG4 KO and WT mice at day 3 (*n* = 4.5), day 6 (*n* = 5.6) and day 16 (*n* = 4.3) post-injection of MC38-Thy1.1 cancer cells. (**C**) Percentage of immune populations within all leukocytes in peritoneal lavages of MC38-Thy1.1 metastasis-bearing VSIG4 KO and WT mice at day16 (*n* = 4.3) post-injection of MC38-Thy1.1 cancer cells. (**D**) Percentage of MC38 Thy1.1^+^ cancer cells in the omental nodules of VSIG4 KO and WT mice at day 6 (*n* = 6.3) and day 16 (*n* = 4.3) post-injection of MC38-Thy1.1 cancer cells. (**E**) Percentage of LPM-like TAMs within all leukocytes in omental tissue bearing MC38-Thy1.1 nodules in VSIG4 KO and WT mice at day 6 (*n* = 6.3) and day 16 (*n* = 4.3) post-injection of MC38-Thy1.1 cancer cells. (**F**) Percentage of immune populations within all leukocytes in omental tissue bearing MC38-Thy1.1 nodules in VSIG4 KO and WT mice at day 6 (*n* = 6.3) and day 16 (*n* = 4.3) post-injection of MC38-Thy1.1 cancer cells. * *p* ≤ 0.05.

Subsequently, we explored the role of the VSIG4 receptor on LPMs in the peritoneal cavity in the context of peritoneal metastasis by colorectal cancer cells. LPMs represent a first line of defense against gastrointestinal pathogens and malignancies, and could, hence, be of importance in the response against peritoneal metastases [39,40]. Hereto, MC38-Thy1.1 cells were injected intraperitoneally in VSIG4 KO and WT mice and the percentage of Thy1.1^+^ cancer cells in the peritoneal cavity was measured at different timepoints via flow cytometry (Gating strategy in Figure S9). No significant differences could be observed between VSIG4 KO and WT mice (Figure 9A). The abundance of LPMs within CD45^+^ cells was also similar in VSIG4 KO and WT mice and equally decreased in both mouse strains as metastasis progressed (Figure 9B). Other immune cell types were also present in equal abundances in both mouse strains at day 16 post-inoculation (Figure 9C).

Since the omentum is often a metastatic niche for cancer cells from gastrointestinal tumors [41,42,43], omental tissues of VSIG4 KO and WT mice were analyzed upon MC38 Thy1.1 intraperitoneal injection. As soon as 6 days post-cancer cell injection, metastatic nodules were visible in the omentum and tumor load was similar between WT and VSIG4 KO mice (Figure 9D). However, by day 16, an increased abundance of MC38-Thy1.1 cancer cells could be observed in VSIG4 WT mice as compared to VSIG4 KO mice, suggesting that the VSIG4 receptor may support metastasis to the omentum (Figure 9D). The abundance of LPM-like TAMs (ICAM2^+^), as well as the immune composition, was similar in the omental tissue bearing tumor nodules from VSIG4 KO and WT mice (Figure 9E,F). Altogether, the VSIG4 receptor does not seem to play a crucial role in the support of metastatic growth of colorectal cancer cells in the liver and the peritoneal cavity, while it may be involved in omental metastasis.

## 4. Discussion

The VSIG4 receptor has been proposed as a potential immune checkpoint, suggesting that its expression may correlate with the progression of cancer. Accordingly, a high expression of *VSIG4* mRNA in tumors correlated with a worse prognosis for CRC patients. In these tumors, *VSIG4* was mainly expressed by macrophages, with the FOLR2^+^ and LYVE1^+^ TAMs being the highest expressors. These data highlight that the VSIG4-expressing TAMs are likely tumor-promoting, but do not necessarily imply a functional role for the VSIG4 receptor. Therefore, we assessed the role of the VSIG4 receptor during the progression of solid CRC and TNBC tumors and CRC liver and peritoneal metastasis in the mouse. VSIG4 mRNA and protein turned out to be absent within murine primary CRC and TNBC tumors, and was redundant for liver and peritoneal metastasis, with the possible exception of omental metastasis.

Several reasons might account for the lack of a difference in primary tumor growth and metastasis in VSIG4 KO and WT mice. A first possibility is the poor translatability of the human VSIG4 receptor to its murine ortholog. In humans, two VSIG4 isoforms exist that either code for a longer receptor with an extracellular IgV and IgC domain, or a shorter receptor with only a single IgV domain [44]. The murine ortholog of VSIG4 encodes for a single shorter receptor, sharing 78% sequence identity with the human shorter isoform [7]. It is possible that the longer VSIG4 isoform in humans is involved in cancer progression and metastasis, rather than the shorter isoform, which could explain the lack of phenotype in mice. Indeed, the longer VSIG4 isoform was more abundantly present in cancerous human tissue, while the shorter isoform was present in healthy human tissue [45]. Contrary to the investigated murine cancer models, the VSIG4 receptor is present as a soluble protein in the blood [18] and expressed in tumor tissue of cancer patients [17,20]. Therefore, it is possible that the longer VSIG4 receptor might play a more dominant role in human cancers and that murine models are coming short for investigating the function of the VSIG4 receptor in cancer.

Secondly, while most TAMs in mouse models are monocyte-derived, a significant fraction of TAMs in human tumors are derived from tissue-resident macrophages. While it is possible for monocyte-derived macrophages to acquire tissue-resident surface receptors [46,47], this may not be equally true in all tissue environments. At the molecular level, it is possible that the murine TME does not provide the relevant signals to upregulate the expression of the VSIG4 receptor or, conversely, provides signals that negatively regulate its expression. For example, the VSIG4 receptor is upregulated by human peripheral blood monocytes when incubated with steroidal anti-inflammatory drugs (dexamethasone) and downregulated when incubated with pro-inflammatory mediators (IFNγ, arachidonate, PMA) [47]. The VSIG4 receptor is also downregulated on KCs in hepatocellular cancer patients that contracted a chronic hepatitis B infection [19], but also on murine LPMs when stimulated with thioglycolate [7], suggesting that pro-inflammatory mediators in the TME could downregulate VSIG4 on TAMs. In this study, E0771 and MC38 tumors were collected at end point, when a volume of 1500 mm^3^ was reached. It is possible that VSIG4^+^ cells could be present in the TME at earlier timepoints, potentially at time points with lower levels of cancer-associated inflammation.

As the VSIG4 protein was completely absent in the TME of subcutaneous MC38 and orthotopic E0771 tumors, a lack of phenotype in VSIG4 KO mice could be anticipated. Of note, the VSIG4 receptor was expressed by resting, tissue-resident macrophages (KCs and LPMs) in tumor-bearing mice, excluding technical issues for detecting the receptor.

VSIG4 expression was detected on tissue macrophages in distant organs, like liver KCs and a subset of resident large peritoneal macrophages. Due to the strategic location of LPMs and KCs, as well as the innate function of the VSIG4 receptor as a phagocytic receptor, we investigated a murine model of CRC metastasis to the liver and the peritoneal cavity. The metastatic burden in the liver and liver immune composition in VSIG4 KO mice was similar as compared to WT control mice, suggesting that VSIG4 is not essential during the early onset and further development of metastatic foci in the liver. While the VSIG4 receptor on KCs is known to capture bloodborne pathogens [8,10], it may not be suited for capturing host-derived cancer cells from the bloodstream. Moreover, the presence of other phagocytic or complement receptors (CR3) might compensate for the lack of the VSIG4 receptor in VSIG4 KO mice [48].

While experimental metastasis to the liver involves a rapid cancer-cell dissemination via the bloodstream, during peritoneal metastasis cancer-cell dissemination occurs rather passively via fluids in the peritoneal cavity. Nevertheless, the immune composition and the seeding of cancer cells in peritoneal lavages were similar in VSIG4 KO and WT mice. The presence of cancer cells in the peritoneal lavage may precede subsequent macroscopic metastasis [49], suggesting that VSIG4 does not contribute to the early events of peritoneal metastasis. However, VSIG4 did seem to increase the macroscopic metastatic burden in the omentum at 16 days post-injection of cancer cells. The omentum is a known premetastatic niche for peritoneal metastasis, and macroscopic omental metastases are associated with a worse prognosis for ovarian cancer patients [41,50]. Moreover, soluble VSIG4 in the peritoneal cavity or ascites might play an immunomodulatory role during inflammation in the peritoneal cavity [20,35,51]. Therefore, it is possible that VSIG4 might facilitate the growth of seeded and established cancer cells in the omentum. Nevertheless, this conclusion might be underpowered considering the low number of biological replicates and further work is warranted to support it.

## 5. Conclusions

Overall, our analysis shows the potential of VSIG4 as a marker for pro-tumoral TAMs in human cancers. Nevertheless, studying its role in TAMs was not achievable in murine tumor models due to the lack of conservation amongst those species. Therefore, caution must be practiced when extrapolating results of murine VSIG4 studies in preclinical cancer research. Nevertheless, other approaches need to be implemented to investigate the role of VSIG4 in TAMs, such as patient-derived xenografts in humanized mice. On the other hand, VSIG4 was detected on some murine tissue-resident macrophages, but metastasis to those organs was unaffected by the absence of VSIG4, suggesting that VSIG4 is redundant in such a setting. However, it remains to be tested whether VSIG4 on tissue-resident macrophages could play a role in primary tumor development in those organs.

## Data Availability

The data generated during the current study are available from the corresponding author upon reasonable request.

## Supplementary Materials

The following supporting information can be downloaded at: https://www.mdpi.com/article/10.3390/cancers17193207/s1, Figure S1: Transcriptomic analysis of the different cell populations infiltrating TNBC and CRC tumors. (A) Dot plot showing the marker gene expression of the different cell clusters infiltrating TNBC tumors. (B) Dot plot showing the marker gene expression of the different cell clusters infiltrating CRC tumors. The color intensity in the dot plot indicates the average gene expression, while the dot size represents the percentage of cells expressing the gene; Figure S2: Transcriptomic analysis of the different TAM populations infiltrating TNBC and CRC tumors. (A) Dot plot of the marker gene expression of the different TAM clusters infiltrating TNBC tumors. (B) Dot plot of the expression of marker genes of the different TAM clusters infiltrating CRC tumors. The color intensity in the dot plot indicates the average gene expression, while the dot size represents the percentage of cells expressing the gene; Figure S3: Kaplan–Meier curves for overall survival of CRC and TNBC patients with high (red) and low (black) VSIG4 expression levels, obtained using https://KMplot.com; Figure S4: Transcriptomic analysis of the different cell populations infiltrating MC38 and E0771 tumors (A) Dot plot of the marker gene expression of the different CD45^+^ clusters infiltrating MC38 tumors. (B) Dot plot of the marker gene expression of the different CD45^+^ clusters infiltrating E0771 tumors. The color intensity in the dot plot indicates the average gene expression, while the dot size represents the percentage of cells expressing the gene; Figure S5: Gating strategy used for the identification of immune populations in MC38 tumors. A representative MC38 tumor in a VSIG4 WT mouse was used to make this gating strategy; Figure S6: VSIG4 is expressed by some tissue-resident macrophages. (A) Fluorescence histograms of VSIG4 expression by TAMs (left 2 panels) and all live cells (right two panels) in E0771 tumors of VSIG4 WT (black) and VSIG4 KO (red) mice. Surface expressed VSIG4 was assessed via an extracellular antibody staining (top 2 panels) while cytoplasmic VSIG4 was assessed via an intracellular staining (bottom 2 panels). A concatenated histogram of *n* = 9/group mice is depicted. (B) Percentage of VSIG4^+^ LPMs and KCs within the macrophage population in the peritoneal lavage and livers, respectively, of VSIG4 WT naïve (*n* = 4, 9) or MC38-bearing mice (*n* = 4, 4); Figure S7: Uncropped blots for Figure 7C; Figure S8: Gating strategy used for the identification of liver immune populations. A representative naïve VSIG4 WT mouse was used to make this gating strategy; Figure S9: Gating strategy to identify immune populations in peritoneal lavages and omental MC38 tumor nodules. A representative VSIG4 WT mouse was used to make this gating strategy; Table S1: Flow cytometry antibody list.
