# Supplementary material for: VSIG4 Is Dispensable for Tumor Growth and Metastasis in Murine Colorectal and Breast Cancer Models
Source: Cancers (Basel). 2025 Oct 1;17(19):3207. doi: 10.3390/cancers17193207 (PMC12523294; doi:10.3390/cancers17193207)
Supplement: Supplementary file 1 [file cancers-17-03207-s001.zip › cancers-3861263-supplementary.pdf]

Supplementary Materials:

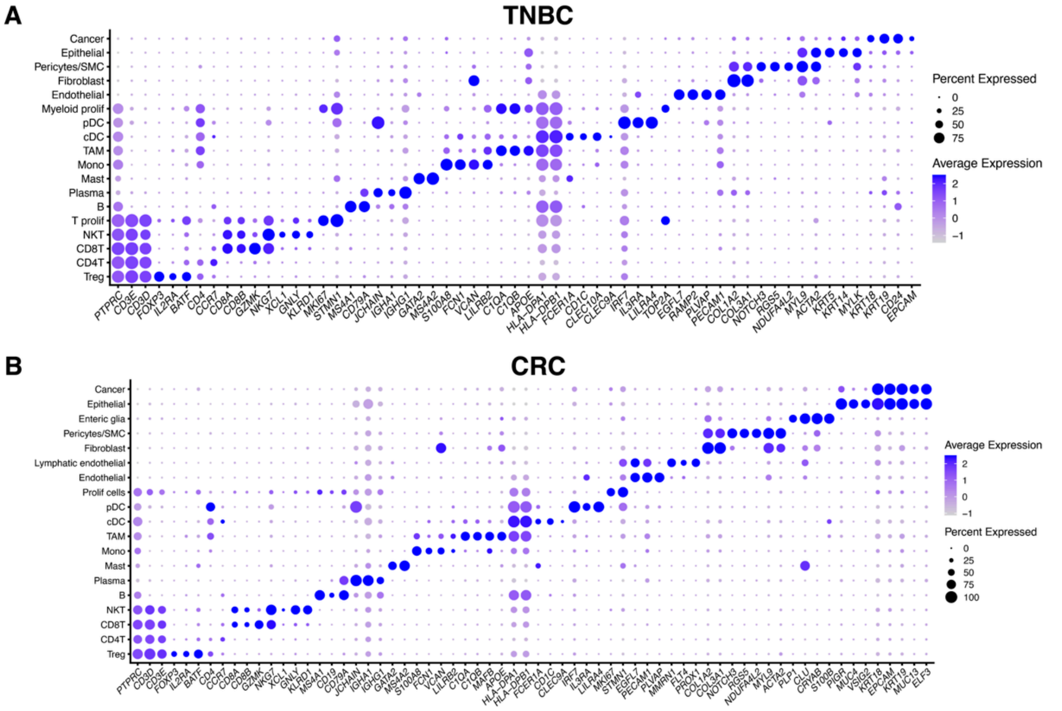

Figure S1: Transcriptomic analysis of the different cell populations infiltrating TNBC and CRC tumors. **(A)** Dot plot showing the marker gene expression of the different cell clusters infiltrating TNBC tumors. **(B)** Dot plot showing the marker gene expression of the different cell clusters infiltrating CRC tumors. The color intensity in the dot plot indicates the average gene expression, while the dot size represents the percentage of cells expressing the gene.

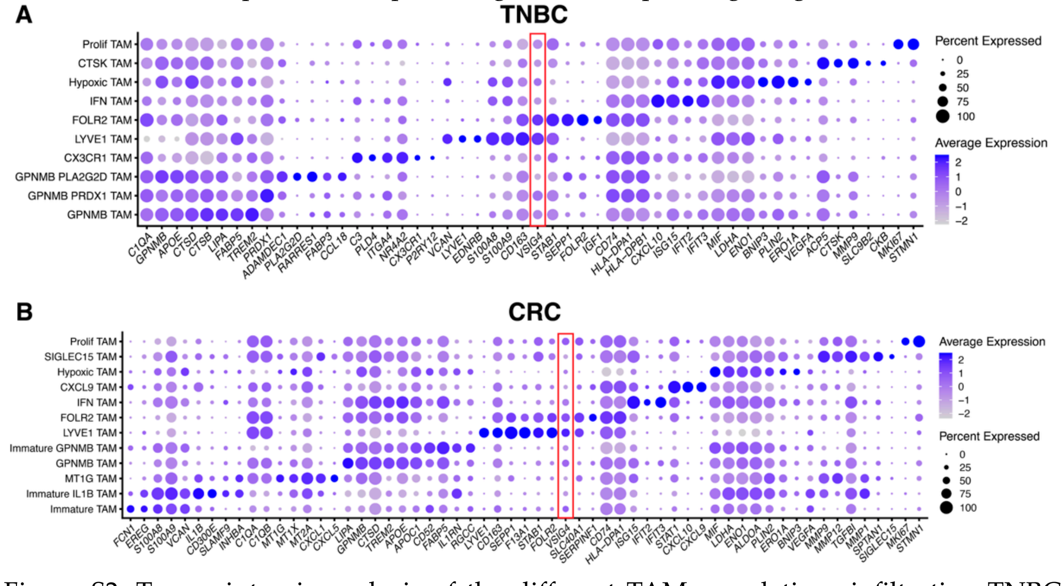

Figure S2: Transcriptomic analysis of the different TAM populations infiltrating TNBC and CRC tumors. **(A)** Dot plot of the marker gene expression of the different TAM clusters infiltrating TNBC tumors. **(B)** Dot plot of the expression of marker genes of the different TAM clusters infiltrating CRC tumors. The color intensity in the dot plot indicates the average gene expression, while the dot size represents the percentage of cells expressing the gene.

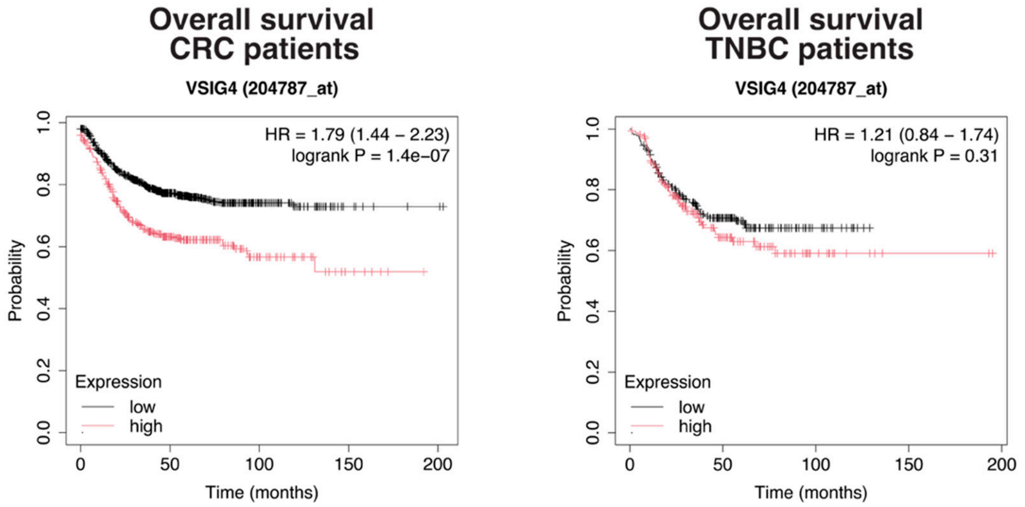

Figure S3: Kaplan Meier curves for overall survival of CRC and TNBC patients with high (red) and low (black) VSIG4 expression levels, obtained using KMplot.com.

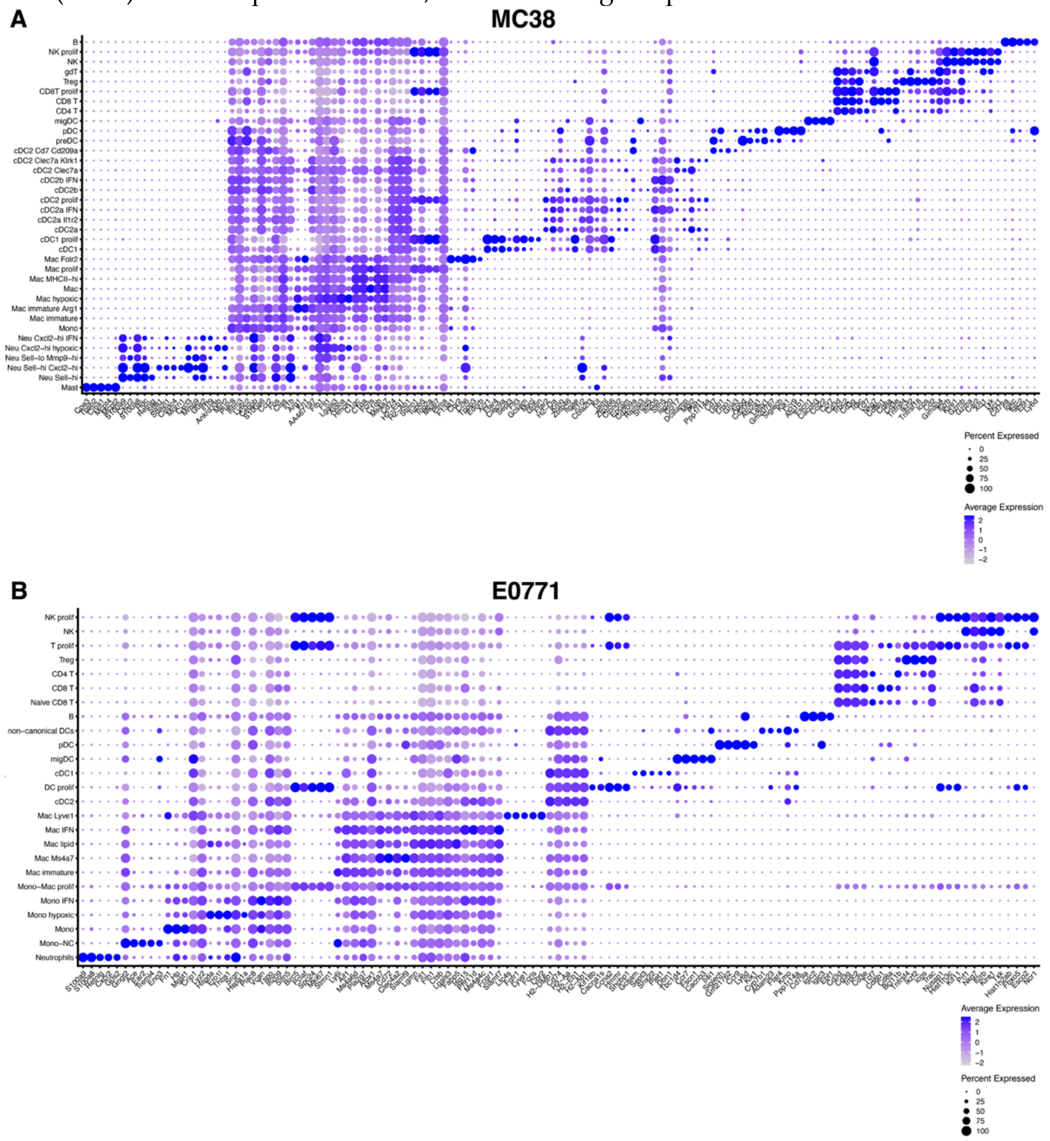

Figure S4: Transcriptomic analysis of the different cell populations infiltrating MC38 and E0771 tumors **(A)** Dot plot of the marker gene expression of the different CD45+ clusters infiltrating MC38 tumors. **(B)** Dot plot of the marker gene expression of the different CD45+ clusters infiltrating E0771 tumors. The color intensity in the dot plot indicates the average gene expression, while the dot size represents the percentage of cells expressing the gene.

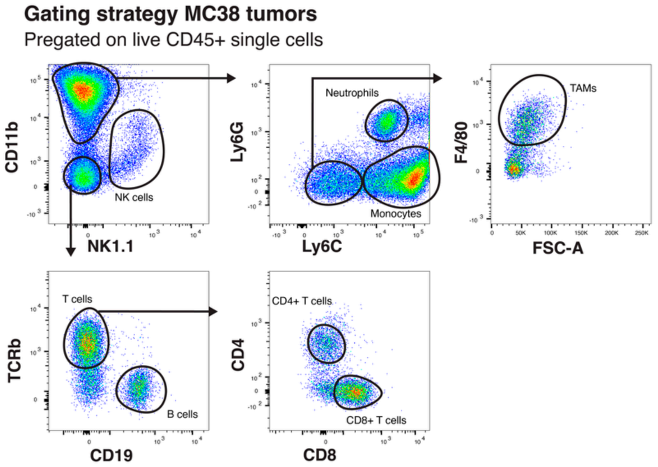

Figure S5. Gating strategy used for the identification of immune populations in MC38 tumors. A representative MC38 tumor in a VSIG4 WT mouse was used to make this gating strategy.

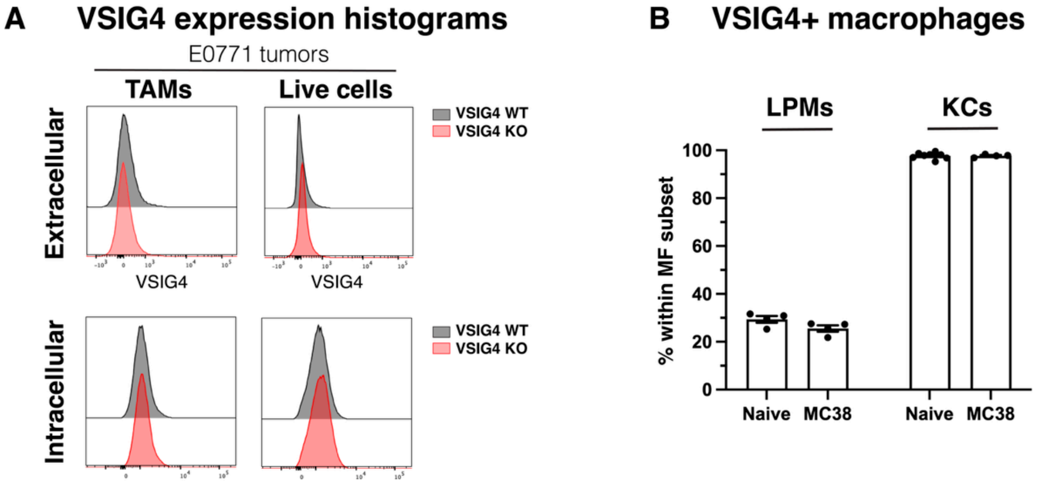

Figure S6. VSIG4 is expressed by some tissue-resident macrophages. **(A)** Fluorescence histograms of VSIG4 expression by TAMs (left 2 panels) and all live cells (right two panels) in E0771 tumors of VSIG4 WT (black) and VSIG4 KO (red) mice. Surface expressed VSIG4 was assessed via an extracellular antibody staining (top 2 panels) while cytoplasmic VSIG4 was assessed via an intracellular staining (bottom 2 panels). A concatenated histogram of $n = 9$/group mice is depicted. **(B)** Percentage of VSIG4$^+$ LPMs and KCs within the macrophage population in the peritoneal lavage and livers, respectively, of VSIG4 WT naïve ($n = 4, 9$) or MC38-bearing mice ($n = 4, 4$).

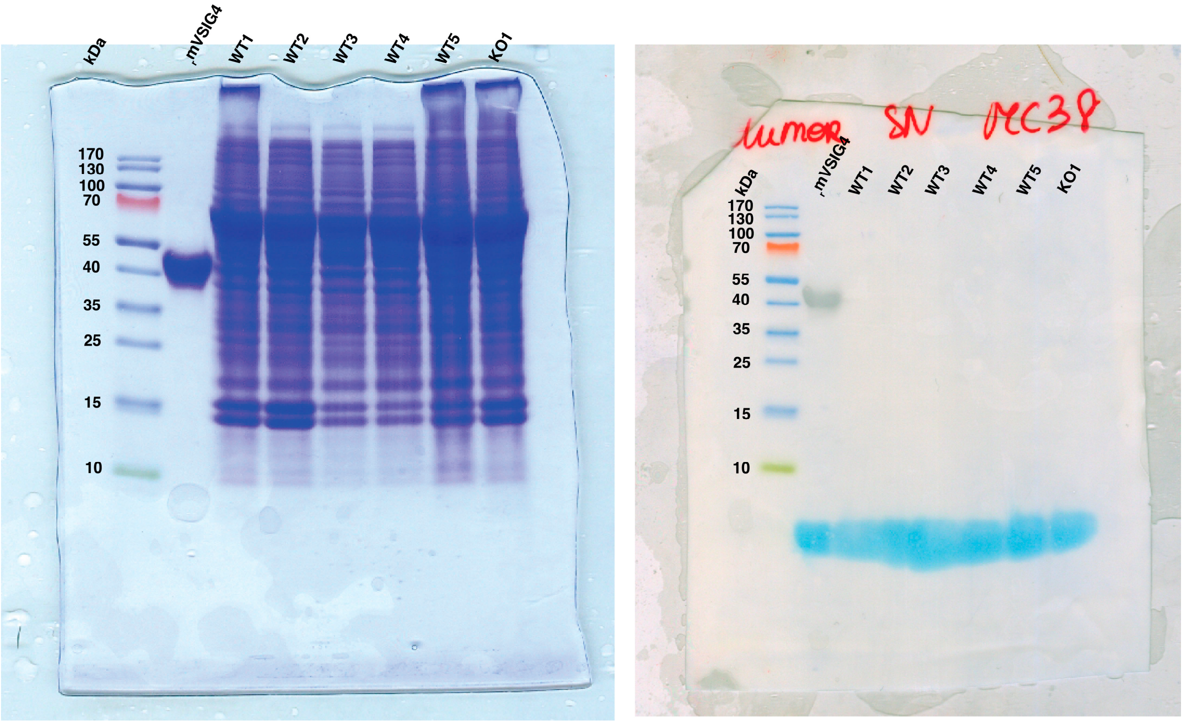

Figure S7. Uncropped blots for Figure 7C

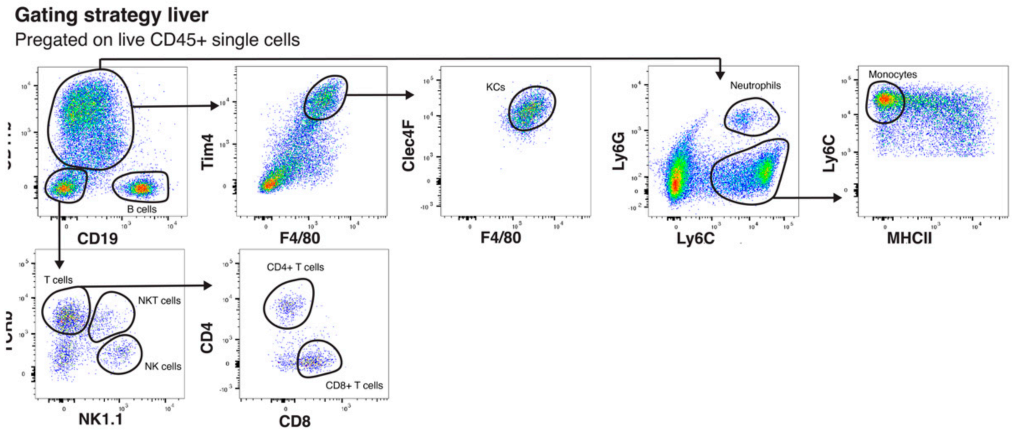

Figure S8. Gating strategy used for the identification of liver immune populations. A representative naïve VSIG4 WT mouse was used to make this gating strategy.

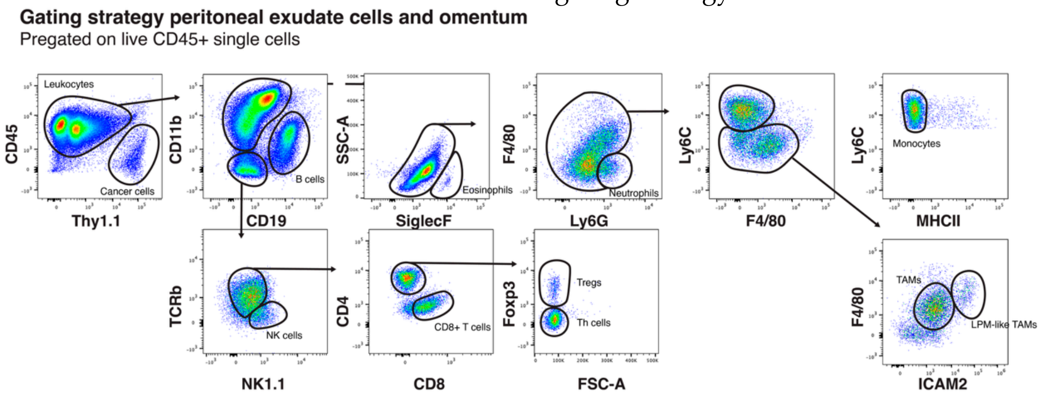

Figure S9. Gating strategy to identify immune populations in peritoneal lavages and omental MC38 tumor nodules. A representative VSIG4 WT mouse was used to make this gating strategy.

Table S1. Flow cytometry antibody list.

Target	Clone	Supplier	Reactivity
CD45	30-F11	Biolegend	Mouse
CD11b	M1/70	Biolegend	Mouse
F4/80	BM8	Biolegend	Mouse
CD102	3C4	Thermofischer	Mouse
MHCII	M5/114.15.2	Biolegend	Mouse
VSIG4	NLA14	Thermofischer	Mouse
Ly6C	HK1.4	Biolegend	Mouse
Ly6G	1A8	Biolegend	Mouse
SiglecF	E50-2440	BD Biosciences	Mouse
CD8	53-6.7	BD Biosciences	Mouse
NK1.1	PK136	eBioscience	Mouse
TCRb	H57-597	eBioscience	Mouse
CD4	GK1.5	eBioscience	Mouse
CD19	1D3	BD Biosciences	Mouse
Foxp3	FJK-16s	eBioscience	Mouse
CD11c	N418	Biolegend	Mouse
Thy1.1	OX-7	BD Biosciences	Mouse
Rat IgG2a kappa	eBR2a	Thermofischer	Rat
Clec4F	3E3F9	Biolegend	Mouse
Tim4	RMT4-54	Biolegend	Mouse
